# SPD_1495 Contributes to Capsular Polysaccharide Synthesis and Virulence in *Streptococcus pneumoniae*

**DOI:** 10.1128/mSystems.00025-20

**Published:** 2020-02-25

**Authors:** Yun-Dan Zheng, Ying Pan, Ke He, Nan Li, Donghong Yang, Gao-Fei Du, Ruiguang Ge, Qing-Yu He, Xuesong Sun

**Affiliations:** a Key Laboratory of Functional Protein Research of Guangdong Higher Education Institutes, Institute of Life and Health Engineering, College of Life Science and Technology, Jinan University, Guangzhou, China; b State Key Laboratory of Biocontrol, College of Life Sciences, Sun Yat-Sen University, Guangzhou, China; Princeton University

**Keywords:** *Streptococcus pneumoniae*, capsule, virulence

## Abstract

Streptococcus pneumoniae, a Gram-positive human pathogen, causes a series of serious diseases in humans. SPD_1495 from S. pneumoniae is annotated as a hypothetical ABC sugar-binding protein in the NCBI database, but there are few reports on detailed biological functions of SPD_1495. To fully study the influence of SPD_1495 on bacterial virulence in S. pneumoniae, we constructed a deletion mutant (D39Δ*spd1495*) and an overexpressing strain (D39*spd1495+*). Comparative analysis of iTRAQ-based quantitative proteomic data of the wild-type D39 strain (D39-WT) and D39Δ*spd1495* showed that several differentially expressed proteins that participate in capsular polysaccharide synthesis, such as Cps2M, Cps2C, Cps2L, Cps2T, Cps2E, and Cps2D, were markedly upregulated in D39Δ*spd1495*. Subsequent transmission electron microscopy and uronic acid detection assay confirmed that capsular polysaccharide synthesis was enhanced in D39Δ*spd1495* compared to that in D39-WT. Moreover, knockout of *spd1495* resulted in increased capsular polysaccharide synthesis, as well as increased bacterial virulence, as confirmed by the animal study. Through a coimmunoprecipitation assay, surface plasmon resonance, and electrophoretic mobility shift assay, we found that SPD_1495 negatively regulated *cps* promoter expression by interacting with phosphorylated ComE, a negative transcriptional regulator for capsular polysaccharide formation. Overall, this study suggested that SPD_1495 negatively regulates capsular polysaccharide formation and thereby enhances bacterial virulence in the host. These findings also provide valuable insights into understanding the biology of this clinically important bacterium.

**IMPORTANCE** Capsular polysaccharide is a key factor underlying the virulence of Streptococcus pneumoniae in human diseases. Thus, a deep understanding of capsular polysaccharide synthesis is essential for uncovering the pathogenesis of S. pneumoniae infection. In this study, we show that protein SPD_1495 interacts with phosphorylated ComE to negatively regulate the formation of capsular polysaccharide. Deletion of *spd1495* increased capsular polysaccharide synthesis and thereby enhanced bacterial virulence. These findings further reveal the synthesis mechanism of capsular polysaccharide and provide new insight into the biology of this clinically important bacterium.

## Abstract (precis)

Capsular polysaccharide is a key factor underlying the virulence of Streptococcus pneumoniae in human diseases. Thus, a deep understanding of capsular polysaccharide synthesis is essential for uncovering the pathogenesis of S. pneumoniae infection. In this study, we show that protein SPD_1495 interacts with phosphorylated ComE to negatively regulate the formation of capsular polysaccharide. Deletion of *spd1495* increased capsular polysaccharide synthesis and thereby enhanced bacterial virulence. These findings further reveal the synthesis mechanism of capsular polysaccharide and provide new insight into the biology of this clinically important bacterium.

## INTRODUCTION

Streptococcus pneumoniae is a major pathogenic bacterium that causes various serious diseases in humans worldwide and may lead to high morbidity and mortality, especially among young children and elderly (1–3). The capsular polysaccharide (CPS) is recognized as one of the most important virulence factors of S. pneumoniae for infecting the host because nonencapsulated bacteria are almost completely harmless (4–6). The ability to regulate CPS production is crucial for the survival of S. pneumoniae in different hosts.

CPS is required for effective colonization in the nasopharyngeal tract of the host and invasive infections in the blood and lungs (6, 7). First, to colonize the pharynx nasalis, S. pneumoniae reduces the synthesis of CPSs to increase the exposure of pneumococcal surface structures, such as adhesins, which are necessary for initial colonization. When S. pneumoniae escapes the nasopharynx, it invades the lungs and subsequently invades the bloodstream. During invasion, CPS is highly expressed to mask the surface antigens in order to reduce complement deposition and protect the bacterium against opsonophagocytosis (4, 8, 9).

Until now, more than 90 antigenically distinct serotypes of S. pneumoniae CPS have been identified, most CPSs are comprised of the repeating units such as glucose (Glu), l-rhamnose (Rha), l-rhamnose, l-rhamnose, glucose, and glucuronic acid (GlcUA), and all the CPSs, except for serotypes 3 and 37, are synthesized in the Wzx/Wzy-dependent pathway (6, 10–12). The *cps* gene cluster participated in the formation of CPS; it is located at the same region of the chromosome between the *dexB* and *aliA* genes, which encodes *cps2E*, *cps2D*, *cps2L*, *cps2I*, etc. (10, 13–15). Further, several possible transcriptional regulators of the *cps* locus, such as RegM (16), ComX (17), CopY (18), CpsR (19), GlnR (20), RitR (21), and ComE (9), have also been reported to regulate CPS formation. In particular, ComE exists in S. pneumoniae in nonphosphorylated and phosphorylated forms, and only the latter form can negatively regulate the expression of the *cps* gene cluster via interaction with the upstream promoter of *cps in vivo* (9).

Our group focused on the relationship between iron transportation and bacterial virulence. We found that in the *ΔpiuA* Δ*piaA* Δ*pitA* triple mutant, in which three major iron transporters were simultaneously deleted, the protein SPD_1495 was evidently upregulated by 6.435- and 86.55-fold at the mRNA and protein levels, respectively, compared to that in D39-WT (22, 23). We speculated that SPD_1495 may also contribute to bacterial virulence. SPD_1495 is annotated as a sugar-binding protein in the NCBI database and is highly conserved in *Streptococcus* species. However, there are few reports on the detailed biological functions and the effects of SPD_1495 on bacterial virulence. Therefore, this study explores the biological function of SPD_1495 in S. pneumoniae using quantitative proteomics combined with biochemical validations. All of the experimental data indicated that SPD_1495 can interact with phosphorylated ComE to negatively regulate CPS formation. Our study provides novel insight regarding CPS formation, as well as the virulence ability of S. pneumoniae.

## RESULTS AND DISCUSSION

### Deletion of *spd1495* results in low utility of sugar source in *S. pneumoniae*.

**FIG 1:**
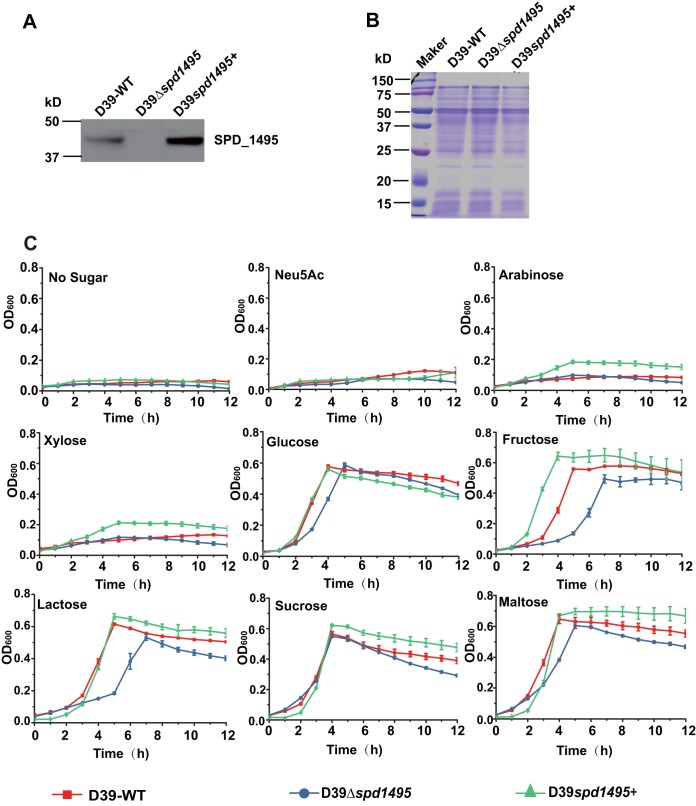
Deletion of *spd1495* affected the growth of S. pneumoniae D39 in medium containing different sugar sources. (A) Confirmation of gene deletion and overexpression of *spd1495* using Western blotting. (B) SDS-PAGE results of whole-cell lysates of D39-WT, D39Δ*spd1495*, and D39*spd1495+* strains as loading controls for the Western blotting experiments of panel A. (C) Growth curves of D39-WT (red), D39Δ*spd1495* (blue), and D39*spd1495*+ (green) strains cultured in C+Y medium containing no sugar, Neu5Ac, arabinose, xylose, glucose, fructose, lactose, sucrose, and maltose. Statistical analysis was conducted using Prism 6.0.

To elucidate the detailed biological function of SPD_1495 in S. pneumoniae, homologous replacement was used to obtain the deletion mutant D39Δ*spd1495*, and the plasmid pIB169-*spd1495* was transformed into D39-WT to construct the overexpression strain D39*spd1495*+. Western blot analysis showed that the expression of SPD_1459 in D39*spd1495*+ was significantly higher than that in D39-WT and that D39Δ*spd1495* hardly expressed SPD_1495. These results indicated that the mutant strains were successfully constructed (Fig. 1A and B).

Since SPD_1495 is annotated as an ABC sugar-binding protein in the NCBI database, the utility of sugar source of D39-WT and D39Δ*spd1495* was detected. Nutrition-restricted C+Y medium with or without a sugar source was used to obtain bacterial growth curves of the D39-WT and mutant strains (Fig. 1C). Without any sugar source in C+Y medium, the D39-WT, D39Δ*spd1495*, and D39-WT+*spd1495* strains almost did not grow. Therefore, *N*-acetylneuraminic acid (Neu5Ac), arabinose, xylose, glucose, fructose, lactose, sucrose, and maltose as the only sugar sources in C+Y medium promoted bacterial growth. The results showed that the addition of Neu5Ac, arabinose, and xylose as the only sugar sources in the culture medium almost rescued the growth of both the WT and mutant strains. The supplementation of glucose, fructose, lactose, sucrose, and maltose as the only sugar sources in C+Y medium can recover cell survival at different levels. Compared to that of D39-WT, D39Δ*spd1495* showed obvious hysteresis of growth, especially when grown in C+Y medium supplemented with fructose and sucrose, whereas D39*spd1495*+ exhibited a marginal increase in growth. These results indicated that SPD_1495 may be a sugar-binding protein in S. pneumoniae and improve sugar utility of bacteria, which is consistent with the annotation in the NCBI database, but its detailed biological function was not reported.

### Deletion of *spd1495* promotes the biosynthesis of CPS.

**FIG 2:**
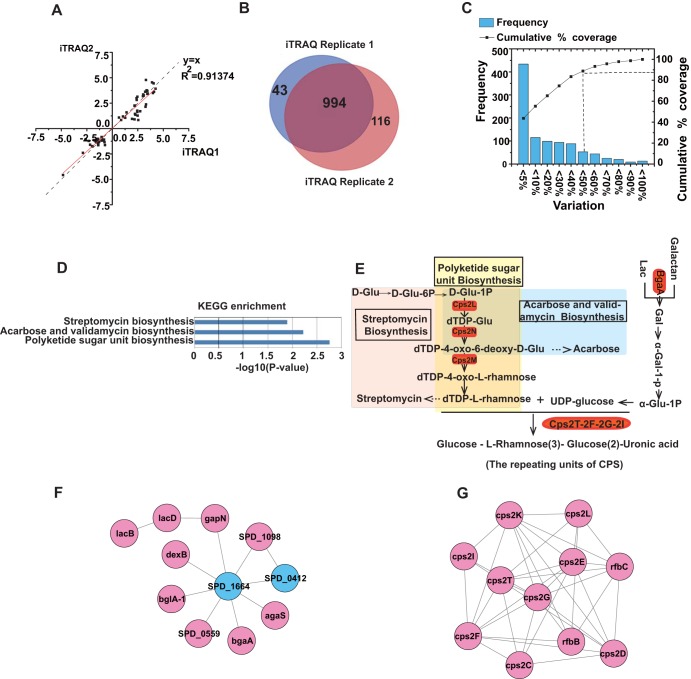
Results of iTRAQ-based proteomics quantitative analysis. (A) Correlation of the fold change in differential proteins in two replicates. (B) The number of total proteins was identified in two biological replicates. (C) Distribution of biological variables in two replicates. (D) KEGG pathway enrichment analysis of differentially expressed proteins. (E) The pathways of polyketide sugar unit biosynthesis, streptomycin biosynthesis, and carbose and validamycin biosynthesis were enriched by KEGG. (F) Differentially expressed proteins involved in sugar metabolism network. (G) Differentially expressed proteins involved in the CPS synthesis network. Red represents upregulated proteins, whereas blue represents downregulated proteins.

To further explore the detailed biological function of SPD_1495 in S. pneumoniae, we used iTRAQ-based proteomics to identify differentially expressed proteins between D39-WT and D39Δ*spd1495*. A strong correlation (*r*^2^ = 0.91374) (Fig. 2A) indicated a good reliability with two independent biological replicates. A total of 994 proteins were commonly detected (Fig. 2B). More than 88% of the proteins had <50% variation (Fig. 2C), and the fold change threshold used to define differentially expressed proteins was <0.67 or >1.50 with *P* < 0.05. The relative quantitative analysis revealed 54 differentially expressed proteins in D39Δ*spd1495*, with 34 upregulated and 20 downregulated proteins, compared to those in D39-WT (see Table S1 in the supplemental material).

10.1128/mSystems.00025-20.1 TABLE S1 Detailed information regarding 54 differentially expressed proteins. Download Table S1, PDF file, 0.02 MB. Copyright © 2020 Zheng et al. 2020 Zheng et al. This content is distributed under the terms of the Creative Commons Attribution 4.0 International license.

**FIG 3:**
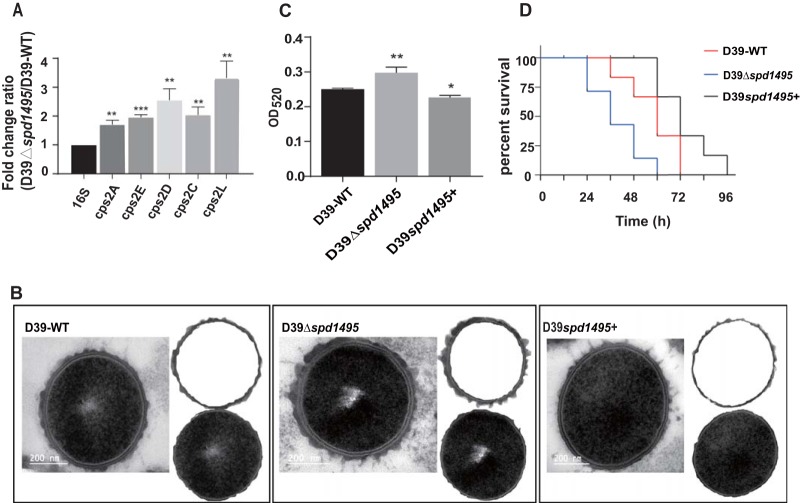
SPD_1495 negatively regulated capsular synthesis. (A) SPD_1495 negative regulate the *cps* genes expression in D39-WT and D39Δ*spd1495.* (B) TEM results for strains D39-WT, D39Δ*spd1495*, and D39*spd1495*+. (C) Uronic acid contents in strains D39-WT, D39Δ*spd1495*, and D39*spd1495*+. (D) The virulence of strains D39-WT, D39Δ*spd1495*, and D39*spd1495*+. Data were analyzed by two-tailed, unpaired Student *t* tests, and results are expressed as means ± the SD. ***, *P* < 0.001; **, *P* < 0.01; *, *P* < 0.05.

KEGG (Kyoto Encyclopedia of Genes and Genomes) pathway enrichment analysis revealed that the differentially expressed proteins were enriched in three biological processes, namely, polyketide sugar unit biosynthesis, streptomycin biosynthesis, and acarbose and validamycin biosynthesis (Fig. 2D and E). It is noteworthy that the biosynthesis of polyketide sugar unit was the most significant pathway with the smallest *P value* among the three pathways. Following deletion of the *spd1495* gene, 34 proteins, including Cps2C, Cps2K, Cps2I, Cps2T, Cps2E and Cps2D etc., which are associated with CPS synthesis were obviously upregulated (Table 1). For example, Cps2L, Cps2M, Cps2N, Cps2T, Cps2F, Cps2G, Cps2I, and BgaA were involved in the synthesis of dTDP–l-rhamnose, a component in CPS synthesis (10). Furthermore, the STRING analysis (Fig. 2F and G) showed that the upregulated proteins in D39Δ*spd1495*, such as Cps2C, Cps2K, Cps2I, Cps2T, Cps2E, and Cps2D, participate in the CPS synthesis network, and the raw materials, such as rhamnose, were used to promote CPS synthesis. The upregulation of *cps* gene was further validated by reverse transcription-quantitative PCR (RT-qPCR) analysis (Fig. 3A). Moreover, we also found that many upregulated proteins in D39Δ*spd1495*, such as LacB, LacD, DexB, BglA-1, SPD_0559, SPD_1664, and SPD_0412, formed a small network. These proteins are involved in carbohydrate metabolism, which supplies some of the key raw materials for CPS synthesis, such as ATP and NAD^+^/NADPH, etc. (24). The 20 downregulated proteins were not enriched into any biological progress. Thus, both KEGG pathway enrichment analysis and STRING analysis indicated that the upregulated proteins are enriched in CPS synthesis. Therefore, we speculated that CPS synthesis in S. pneumoniae is negatively regulated by SPD_1495.

**TABLE 1:** Some differently expressed proteins in D39Δ*spd1495* compared to D39-WT

Gene or protein	Protein	Avg fold change	Avg –log_10_ *P*
Carbohydrate metabolic process			
*bgaA*	β-Galactosidase, putative	4.30	9.80
*bglA-1*	6-Phospho-β-glucosidase	7.55	4.89
*dexB*	Glucan 1,6-α-glucosidase	19.24	4.24
*lacD*	Tagatose 1,6-diphosphate aldolase	2.89	3.48
*lacB*	Galactose-6-phosphate isomerase	3.82	1.88
			
Capsule-associated proteins			
Cps2L	Glucose-1-phosphate thymidylyltransferase	10.86	6.94
Cps2K	Cps2K	14.65	0
Cps2C	Polysaccharide export protein	9.18	4.85
Cps2D	Cps2D	4.66	1.50
Cps2F	Glycosyl transferase, group 2 family protein	10.87	5.70
Cps2G	Glycosyl transferase, group 1 family protein	8.57	2.50
Cps2M	Cps2M	11.96	2.27
Cps2I	Cps2I	7.14	2.26
Cps2T	Cps2T	11.92	6.56
Cps2E	Cps2E	6.15	2.19
Cps2N	dTDP-glucose 4,6-dehydratase	17.01	7.51

To validate the relationship between the CPS synthesis and SPD_1495 in S. pneumoniae, transmission electron microscopy (TEM) was used to detect the thickness of the CPS. The images of TEM showed that the thickness of CPS decreased in the following order: D39Δ*spd1495* > D39-WT > D39*spd1495*+. This indicates that SPD_1495 could negatively regulate CPS formation (Fig. 3B). The relative content of uronic acid, which is the main component of the capsule, was determined to further quantitatively compare the difference in CPS formation. As shown in Fig. 3C, the content of uronic acid decreased in the following order: D39Δ*spd1495* > D39-WT > D39*spd1495*+; this is consistent with the order of decrease in capsule thickness. Therefore, both the thickness of CPS and the content of uronic acid confirmed that, compared to the D39-WT, D39Δ*spd1495* produced higher quantities of CPS. These results jointly indicated that SPD_1495 negatively regulated capsule formation.

### Deletion of *spd495* increases virulence in *S. pneumoniae*.

The CPS plays an important role in the virulence of S. pneumoniae (25). The survival rates of mice infected with D39-WT, D39Δ*spd1495*, and D39*spd1495*+ were determined to uncover the contribution of SPD_1495 to bacterial virulence. As shown in Fig. 3D, the D39Δ*spd1495* mutant was more virulent than D39-WT; however, the virulence of SPD_1495-overexpressing strains was very similar to that of D39-WT in the mouse pneumonia model. This result indicated that SPD_1495 may increase the virulence of S. pneumoniae by decreasing the formation of the CPS and demonstrated that SPD_1495 affects the pathogenicity of S. pneumoniae during infection *in vivo*.

### SPD_1495 affects the formation of *S. pneumoniae* CPS via interacting with phosphorylated ComE.

**FIG 4:**
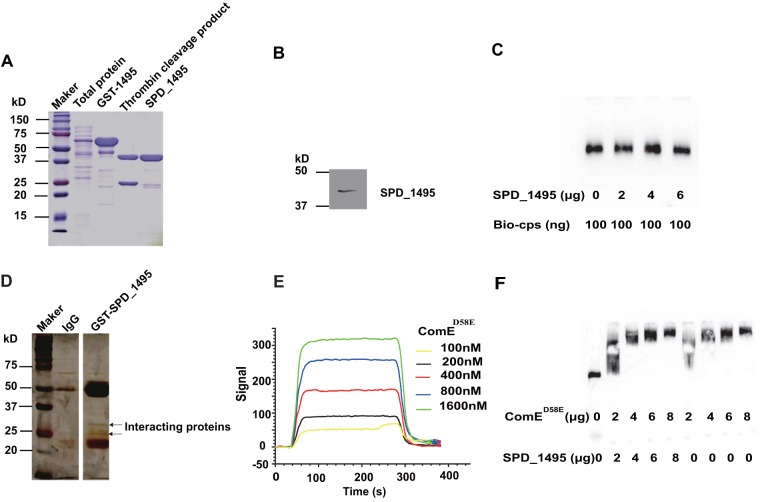
SPD_1495 interacted with ComE. (A) SDS-PAGE results of SPD_1495 expression, visualized using a PGEX-4T-1 fusion system and purified by GST affinity column. (B) Western blotting assay of SPD_1495 expression using anti-SPD_1495 antibody. (C) EMSA with SPD_1495 cannot bind to Bio-*cps* probe. (D) The potential proteins interacting with SPD_1495 were screened by a Co-IP assay and are indicated by arrows. (E) SPR detected the interaction of ComE^D58E^ with SPD_1495. (F) EMSA demonstrated that ComE^D58E^, the complexes of ComE^D58E^, and SPD_1495 bind to the Bio-*cps* probe.

In order to seek regulation network of SPD_1495, the SPD_1495 was expressed and purified by glutathione *S*-transferase (GST) tag affinity chromatography. There was a band of ∼45 kDa, corresponding to the calculated molecular weight of SPD_1495 in SDS-PAGE, the Western blotting with SPD_1495 antibody confirmed the expression of SPD_1495 (Fig. 4A and B). The proteomic data and detection of capsule showed that the formation of CPS was negatively regulated by SPD_1495. In S. pneumoniae, the *cps* gene cluster is mainly responsible for the CPS biosynthesis (19). We speculated that SPD_1495 may regulate the expression of the *cp*s gene cluster via interaction with the CPS operon. However, electrophoretic mobility shift assay (EMSA) results revealed that SPD_1495 cannot directly interact with *cps* operon (Fig. 4C). A previous study by Zheng et al. identified the transcriptional regulator in the upstream promoter region of *cps* by DNA-pulldown but did not find SPD_1495 (9). Therefore, we deduce that SPD_1495 may negatively regulate CPS formation indirectly.

A coimmunoprecipitation (Co-IP) assay was performed to identify the proteins interacting with SPD_1495. Compared to that of IgG in the control group, SPD_1495 specifically captured some proteins of molecular weight between 20 and 37 kDa (Fig. 4D). These proteins were cut and digested with trypsin and then were identified by mass spectrometry. The identification results showed that SPD_1495 can interact with several proteins, including RpsC, ComE, SPD_1495, RecA, CshB, and CoaC (Table 2). Notably, ComE exists in two forms (nonphosphorylated and phosphorylated) in S. pneumoniae, and only phosphorylated form ComE negatively regulates the expression of the *cps* gene cluster via an interaction with the upstream promoter of *cps* (9). Thus, to further uncover the function of SPD_1495 in the formation of CPS, a phosphomimetic form (ComE^D58E^) was expressed and purified to check its interaction with SPD_1495 by surface plasmon resonance (SPR). The result of SPR studies also showed that ComE^D58E^ can interact with SPD_1495 directly *in vitro* (Fig. 4E), and their dissociation constant is 1.55 × 10^−6^ M. Moreover, EMSA showed that ComE^D58E^ interacting with SPD_1495 has a stronger binding ability than ComE^D58E^ on its own for the same Bio-*cps* probe (Fig. 4F), indicating that SPD_1495 contributes to the binding between ComE^D58E^ and the *cps* operon and thus regulates CPS synthesis. Furthermore, RT-qPCR results demonstrated that the expression levels of CPS-related genes were upregulated in D39Δ*spd1495* (Fig. 3A). Moreover, the proteomics data showed the consistent result that many proteins involved in CPS synthesis were upregulated in D39Δ*spd1495*. In summary, our data suggest that SPD_1495 can interact with phosphorylated-ComE in S. pneumoniae to negatively regulate CPS formation.

**TABLE 2:** Proteins interacting with of SPD_1495 identified in a Co-IP assay

Protein	Function	Coverage (%)
RpsC	30S ribosomal protein S3	73.7
ComE	Response regulator	50.4
SPD_1495	Sugar ABC transporter, sugar-binding protein	58.8
RecA	Protein RecA	54.6
CshB	DEAD-box ATP-dependent RNA helicase CshB	58.8
CoaC	Phosphopantothenoylcysteine decarboxylase	53.6

### Amino acid sequence homologous alignment and protein evolutionary tree analysis.

**FIG 5:**
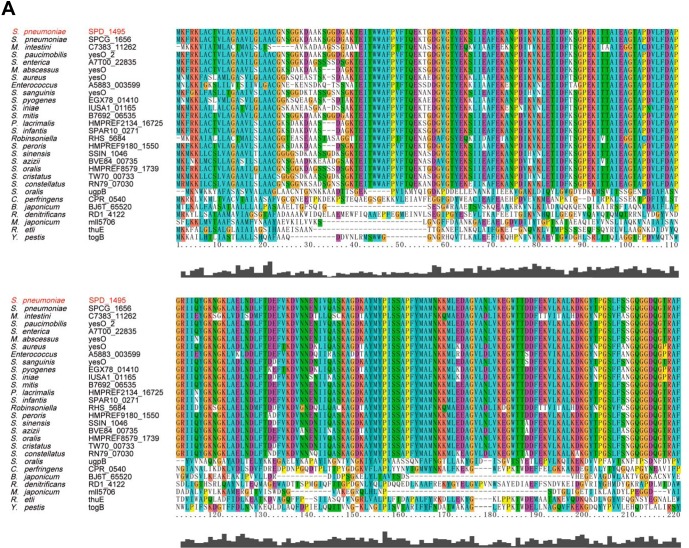

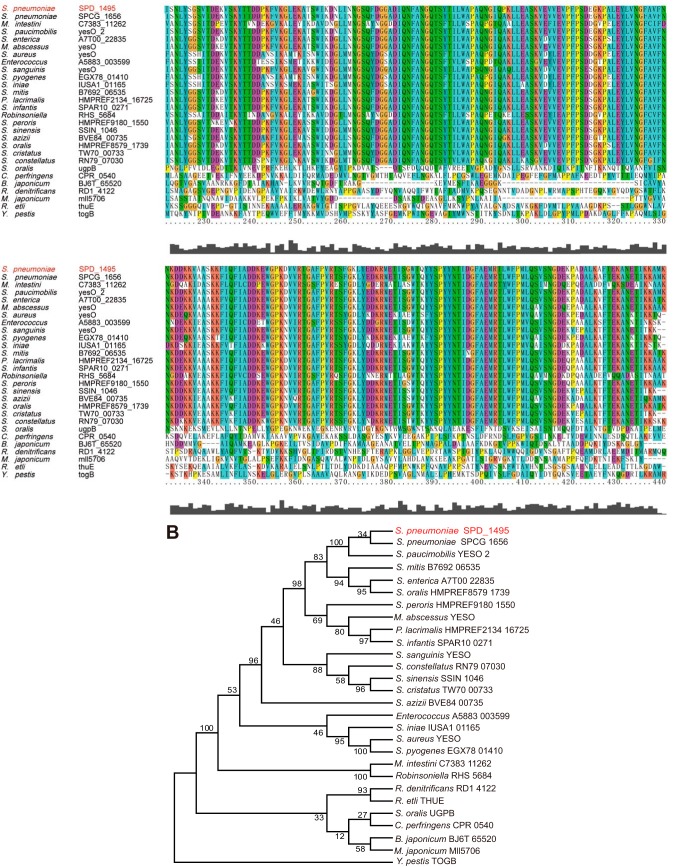
Evolutionary analysis of SPD_1495. (A) Multiple sequence alignment of SPD_1495 with homologous proteins in various bacterial species. (B) Protein evolutionary tree of SPD_1495 and homologous proteins.

To assess the conservation of SPD_1495 in bacteria, amino acid sequence alignment and the phylogenic tree construction were carried out (Fig. 5A and B). The result showed that the SPD_1495 protein is highly conserved in Gram-positive bacteria, especially in the genus *Streptococcus*. A database search showed that several highly homologous proteins of SPD_1495, for example, B7692_06535, YESO_2, and SPAR10_0271 are also annotated as sugar ABC transporter substrate-binding proteins. The detailed biological function of this protein family has not been reported. This is the preliminary report of the function of SPD_1495 in S. pneumoniae CPS synthesis. Hence, the regulation of capsule formation and bacterial virulence of SPD_1495 observed in the present study may represent a novel biological function of this cluster of proteins.

Previous research has shown that the CPS of S. pneumoniae is one of the key virulence factors required for effective infection host (26, 27), and many vaccines targeting CPS have been developed to treat or prevent bacterial infections in recent years (28). In this work, convincing evidence was obtained indicating that SPD_1495 can negatively regulate CPS synthesis by interacting with phosphorylated ComE in S. pneumoniae. Further, SPD_1495 is highly conserved among Gram-positive bacteria. Our data suggest that SPD_1495 is a vaccine or drug target candidate because of its contribution to virulence during bacteremia. Accordingly, our further studies will investigate whether SPD_1495 can be an efficacious vaccine antigen for preventing S. pneumoniae infection.

### Conclusion.

**FIG 6:**
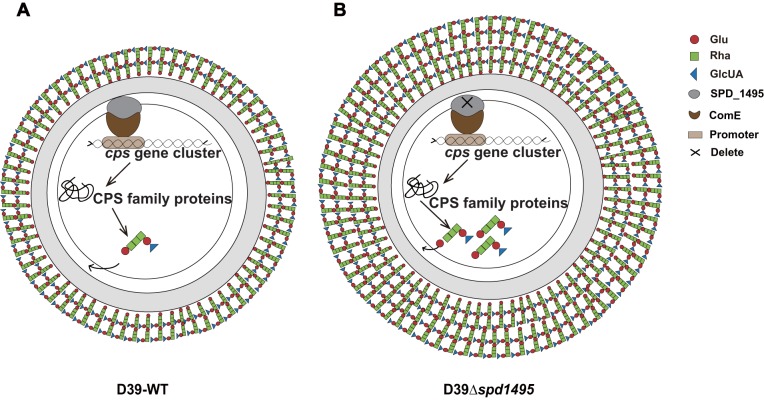
Integrated model of SPD_1495 function in S. pneumoniae in this study. (A) In D39-WT, SPD_1495 interacted with ComE to negatively regulate capsular polysaccharide synthesis, resulting in normal capsule thickness. (B) In D39Δ*spd1495*, without interaction between SPD_1495 with ComE, the bacterial capsule became thickened.

Taken together, our research revealed the biological function of SPD_1495 in capsule formation in S. pneumoniae. This protein negatively regulates CPS synthesis by interacting with phosphorylated ComE, a key transcriptional regulator located in the *cps* locus. Thus, deletion of the *spd1495* gene will increase the production of dTDP–l-rhamnose, thereby promoting the biosynthesis of CPS. Furthermore, the CPS is recognized as one of the most important virulence factors in S. pneumoniae for infecting the host. deletion of *spd1495* will upregulates the CPS to improve the virulence factor related to the host, thereby enhancing the ability of S. pneumoniae to infect host cells (Fig. 6). Considering the importance of CPS in bacterial adherence and entry into the host, regulation of SPD_1495 is essential in *Streptococcus* infection. Moreover, the animal assay indicated that SPD_1495 is an important protein in regulating the virulence in S. pneumoniae. Therefore, SPD_1495 may be a vaccine or drug target candidate for the development of novel antibacterial to treat the *Streptococcus* infections.

## MATERIALS AND METHODS

### Bacterial growth conditions.

The S. pneumoniae D39 was cultured in THY medium (Todd-Hewitt broth; Oxoid, UK) with 0.5% yeast extract (Oxoid) or on Columbia agar (Difco, USA) containing 5% sheep blood (Ruite, China) at 37°C in a 5% CO_2_ incubator (MCO-170AICUVHL-PC; Panasonic, Japan). Escherichia coli DH5ɑ and BL21 (Invitrogen, USA) were both cultured in Luria-Bertani (LB) medium at 37°C in an incubator shaker (Yi Heng, China). All strains were stored at –80°C in 15% glycerol. Erythromycin (Erm; Sigma, USA) at 0.25 mg/ml, chloramphenicol (Cm; Sigma) at 4 mg/ml, or ampicillin (Amp; Sigma) at 100 μg/ml was added to the medium when needed for selective culture. All of the bacterial strains and plasmids used are listed in Table 3.

**TABLE 3:** Bacterial strains and plasmids used in this study

Strain or plasmid	Relevant characteristic(s)^*a*^	Source or reference
Strains		
*S. pneumoniae*		
D39	Wild type	ATCC (USA)
Δ*spd1495* mutant	In-frame *spd1495* mutant strain derived from D39; Erm^r^	This study
D39/pIB169*-spd1495*	D39 strain transformed with pIB169*-spd1495*; Cm^r^	This study
*E. coli*		
BL21	Wild type	Invitrogen (USA)
BL21/pGEX-4T-1*-spd1495*	BL21 strain transformed with pGEX-4T-1*-spd1495*; Amp^r^	This study
		
Plasmids		
pGEX-4T-1	pGEX vector contained *tac* promoter; Amp^r^	Invitrogen (USA)
pGEX-4T-1-*spd1495*	*S. pneumoniae* D39 *spd1495* (33–442 AA) fragment cloned into pGEX-4T-1; Amp^r^	This study
pIB169	Shuttle plasmid contained P*_veg_* promoter; Cm^r^	14
pIB169-*spd1495*	*S. pneumoniae* D39 *spd1495* (1–442 AA) fragment cloned into pIB169; Cm^r^	This study
pET-28b(+)	pET-28b(+) vector contained T7 promoter; Kan^r^	Novagen (USA)
pET-28b(+)*-comE^D58E^*	*S. pneumoniae* D39 *comE* (1–250 AA) fragment cloned into pIB169; Cm^r^	This study

a Erm^r^, erythromycin resistance; Cm^r^, chloramphenicol resistance; Amp^r^, ampicillin resistance. Kan^r^, kanamycin resistance; AA, amino acids.

### Construction of mutant *S. pneumoniae* D39 strains.

Long-flanking-homology PCR (LFH-PCR) was used to construct strain D39Δ*spd1495* (29, 30), and the primers used in this study are listed in Table 4. The target gene, *spd1495* (1,329 bp), was replaced by an antibiotic resistance cassette gene (*erm*). The reconstructed region, including *erm* (829 bp), upstream (817 bp) and downstream (624 bp) of *spd1495* was ligated by LFH-PCR using primers P3 to P8. The products of LFH-PCR were transferred into strain D39-WT, and the positive transformant was selected by Erm-containing Columbia sheep blood agar plate. D39Δ*spd1495* strains were stocked after seven sequential passages in the THY medium with 0.25 μg/ml Erm. To construct *spd1495*-overexpressing strains, the recombinant plasmid pIB169-*spd1495* was constructed by inserting the *spd1495* gene (1,329 bp) into pIB169 plasmid using a ClonExpress II one-step cloning kit (Vazyme, China). The recombinant plasmid pIB169-*spd149*5 containing an Cm resistance cassette was then transferred into the D39-WT strain. The transformants were screened in Cm-containing Columbia sheep blood agar plate. Finally, all of the mutant strains, including D39Δ*spd1495* and D39*spd1495+*, were confirmed by Western blotting (31).

**TABLE 4:** Primers used in this study

Primer	Sequence (5′–3′)
P1(spd1495-F)	GATCTGGTTCCGCGTGGATCCTCAGGTGGTGACGGTGCCAAAACAG
P2(spd1495-R)	TCAGTCAGTCACGATGCGGCCGCCTATTGTTTCATAGCTTTTTTG
P3(spd1495-up-F)	GCCTGTTTAGCGATGTACCATAGTG
P4(spd1495-up-R)	ATTCTATGAGTCGCTGCCGACTTCCTTAGTTATTCTATAAAAAGT
P5(erm-F)	AGTCGGCAGCGACTCATAGAAT
P6(erm-R)	CCGGGCCCAAAATTTGTTTGAT
P7(spd1495-down-F)	ATCAAACAAATTTTGGGCCCGGTTGTATTCTCCTATGTAATAAGC
P8(spd1495-down-R)	GTGGGTCATAAATCCATTTCCAAAC
P9(pIB169-spd1495-F)	GGAGACCGCGGTCCCGAATTCATGAAATTTAGAAAATTAGCTTGTACAGTAC
P10(pIB169-spd1495-R)	GGTCGACCTCGAGGGATCCGTGATGGTGATGGTGATGTTGTTTCATAGCTTTTTTGATTG
P11(ComE-F)	GGATCTTCCAGAGATGGATCCATGAAAGTTTTAATTTTAGAAGATGTTATTGA
P12(ComE-R)	CTGCCGTTCGACGATCTCGAGTCACTTTTGAGATTTTTTCTCTAAAATATC
P13(ComE^D58E^-R)	TTATTTCCTAGAAATCGATATTCATGGAATTGA
P14(ComE^D58E^-F)	GAATATCGATTTCTAGGAAATAAAGCTGATTTA
P15 cpsF Bio-	TACACATCTGCTTCTAAAATATTGT (labeled by biotin)
P16 cpsR	TTAAAACGTCTACTCATGATTAACA
P17 cps2A-F	TAGCCGATGGAGACCGTGAC
P18 cps2A-R	TCTGGCATTGCATAAGAAGGAAG
P19 cps 2C-F	ACGACTCCTTCTTCTCCAAATGTT
P20 cps 2C-R	CAAATCTGGAACGAGCCCTAAA
P21 cps 2D-F	ACCGAATCCGACAGCTCTTCT
P22 cps 2D-R	TCACCTGCCTCCGTCACTAAA
P23 cps 2E-F	TTGTTGGGGAGTTGGTAGCCG
P24 cps 2E-R	TGCCATCTCACGAATTTGCTT
P25 cps 2L-F	GATAGCGTTGCTTTAATCTTGGG
P26 cps 2L-R	GGAGCGAGGACACTCTGGTTTT
P2716S rRNA-F	CTGCGTTGTATTAGCTAGTTGGTG
P2816S rRNA-R	TCCGTCCATTGCCGAAGATTC

### Cloning, expression, and purification of SPD_1495 and ComE^D58E^.

The *spd1495* gene was amplified from S. pneumoniae D39 genomic DNA (the primers are listed in Table 4). The product of PCR and pGEX-4T-1 plasmid were digested by restriction enzymes BamHI and Not I (TaKaRa, Japan), and then the digested fragments were ligated by the ClonExpress II kit. The constructed plasmid pGEX-4T-1-*spd1495* was verified by sequencing (Sangon Biotech, China) and then transferred into E. coli BL21. For SPD_1495 expression, BL21/pGEX-4T-1-*spd1495* was cultured in LB medium with 100 μg/ml Amp in a shaking incubator at 37°C. When the optical density at 600 nm (OD_600_) reached 0.6 to 0.8, protein expression was induced by adding 0.5 mM isopropyl-β-d-thiogalactopyranoside (IPTG; Sigma) with a further 5 h of incubation. The bacteria were harvested by centrifugation at 8,000 × *g* for 10 min at 4°C and washed with 0.01 M phosphate-buffered saline (PBS) three times. The harvested cells were suspended in 0.01 M PBS and lysed by sonication. GST-SPD_1495 fusion protein was isolated by glutathione-Sepharose 4B (GE Healthcare Life Science, Sweden) and digested with thrombin in 0.01 M PBS buffer according to the manufacturer’s instructions to remove the GST tag. The purified SPD_1495 was then used as an antigen for antibody preparation (Tianjin Sun gene Biotech Co., China). The BALB/c mice (6 to 8 weeks old, weighing ∼20 g) were immunized with 100 μg of GST-SPD_1495 by subcutaneous injections. After 2 weeks, secondary immunization was performed. A booster dose was administered intravenously to enhance immunity. The spleen cells of the immunized mice were then fused with hybridoma cells and diluted in Dulbecco modified Eagle medium. Hybridomas were tested by indirect enzyme-linked immunosorbent assay (Cusabio, China). The isotype of each monoclonal antibody (MAb) was determined. Ascites were produced in BALB/c mice, and then MAbs were purified and biotinylated (32). A phosphomimetic mutant, ComE^D58E^, was constructed by site-directed mutagenesis of *comE* to exhibit the activity of ComE protein *in vitro*. Both wild-type ComE and ComE^D58E^ were expressed and purified as described previously (9, 33, 34). The primers are listed in Table 4 (ComE-F to 16S rRNA-R).

### Western blot assay.

From each sample, 25 μg of protein was loaded into 12% SDS-PAGE gels and then transferred onto a polyvinylidene fluoride (polyvinylidene difluoride) membrane (Millipore, USA). Anti-SPD_1495 antibody was incubated with polyvinylidene difluoride membrane at 4°C, and horseradish peroxidase-conjugated goat anti-mouse was used as a secondary antibody. The results were visualized with Clarity Western ECL Substrate (Bio-Rad, USA) and captured using ImageMaster 2D Platinum 6.0 (GE Healthcare, USA). Meanwhile, SDS-PAGE gels stained with Coomassie brilliant blue G250 were used as a loading control.

### Growth curve analysis.

The D39-WT and mutant strains were cultured in C+Y medium (no sugar) with Neu5Ac, arabinose, xylose, glucose, fructose, lactose, sucrose, and maltose as only sugar source, respectively, at equivalent inoculation doses at 37°C in a 5% CO_2_ incubator. The OD_600_ was continuously measured every hour by UV-visible spectroscopy (Evolution 300; Thermo Fisher Scientific, USA) for 12 h, and the data were analyzed by Prism 6.0 (GraphPad Software, USA).

### Protein preparation, iTRAQ labeling, and proteomics analysis.

From each sample, 200 μg of protein was extracted from D39-WT and D39Δ*spd1495* strains in the exponential growth phase, digested with trypsin (Promega, USA) at 37°C for 16 h, and lyophilized. Then, a iTRAQ reagent multiplex kit (AB Sciex, USA) was used to label peptide samples according to the manufacturer’s protocol. D39-WT and D39Δ*spd1495* strains were labeled with 114 and 116 isobaric tags for 1 h at room temperature, respectively. The labeled peptides from the two groups were mixed in equal proportions and dried. High-performance liquid chromatography (WuFeng, China) with a TechMate C18-ST column (4.6 mm × 250 mm, 5 μm; TechMate, China) was used to separate the labeled peptides. In brief, an iTRAQ-labeled peptide mixture was eluted with a gradient of 2 to 80% acetonitrile in 20 mM ammonium formate (pH 10.0) for 65 min at a flow rate of 0.8 ml/min. The peptide elution was monitored at 214 nm. The eluted peptides were collected every minute, pooled into six fractions, and then lyophilized. MS analyses were performed with a TripleTOF 5600 (AB Sciex) mass spectrometer. The acquired raw data files (wiff) of each fraction were combined to search against the protein database of S. pneumoniae D39, and ProteinPilot software 4.5 (AB Sciex) was used to identify and quantify the proteins. The parameters used for mass spectrometry and quantitative analysis were set as follows: sample type, iTRAQ 4plex (peptide labeled); cysteine alkylation, iodoacetic acid; digestion, trypsin; instrument, Triple-TOF 5600; ID focus, biological modifications; database, S. pneumoniae D39_fasta; search effort, thorough; and detected protein threshold [unused ProtScore (Conf)], >1.30 (95.0%) (23, 35). The iTRAQ was then used for protein quantification, and peptides with a global false discovery rate of ≤1% were considered for further analysis. The fold change was calculated as the 116/114 ratio for differentially expressed proteins. In this study, the cutoff threshold for upregulation and downregulation of the differentially expressed proteins were defined using the population statistics applied to the biological replicates reported by Gan et al. (36). The finalized cutoffs for upregulation (>1.5) and downregulation(<0.67) of proteins were used.

KEGG was used for the pathway enrichment analysis of 54 differentially expressed proteins in the “Wu Kong” platform (37, 38). Protein-protein interaction network analysis (score > 0.4) using the STRING (39) database (version 10.5) was performed, and the results were visualized with Cytoscape (v3.5.1) (40). The interaction network was further analyzed using Molecular Complex Detection (MCODE) (41).

### Transmission electron microscopy analysis.

The D39-WT, D39Δ*spd1495*, and D39*spd1495+* strains in the exponential growth phase were harvested by centrifugation at 10,000 × *g* for 5 min at 4°C and washed with 0.01 M PBS for three times. Then, all cell pellets were fixed with 2.5% glutaraldehyde fixative and postfixed in 1% osmium tetroxide for 2 h at 4°C. Subsequently, 2% uranyl acetate was added to dye the cell pellets for 2 h at room temperature. After the dyeing step, the sample was dehydrated with 50% acetone for 15 min, 70% acetone for 15 min, 90% acetone for 15 min, and twice in 100% acetone for 20 min. All samples were then fixed in epoxy resin and left to polymerize at 37°C for 24 h, 45°C for 24 h, and 60°C for 48 h. Each sample was cut into approximately 50- to 80-nm thin slices and then dyed with 3% uranyl acetate and lead citrate. Finally, the prepared samples were detected using TEM (JEOL 2100F; Japanese Electronics Company).

### Determination of uronic acid content.

The D39-WT, D39Δ*spd1495*, and D39*spd1495+* strains in the exponential growth phase were harvested by centrifugation at 8,000 × *g* for 10 min at 4°C and washed with 0.01 M PBS three times. Next, the bacteria were resuspended the in Tris-HCl/MgSO_4_ buffer (Genebase and Sigma, USA) and centrifuged at 8,000 × *g* for 5 min at 4°C to remove the supernatant and then resuspended with 500 μl of Tris-HCl/MgSO_4_ buffer again. Next, 200 μl of bacterial solution and 1.2 ml of 12.5 mM tetraborate solution were mixed and heated at 100°C for 5 min. All of the samples were placed in ice water, and 20 μl of 15% *m*-hydroxybiphenyl solution was added to each sample, followed by thorough mixing. Meanwhile, the same volume 0.5% NaOH was added in control group, Finally, the concentration of each sample was detected in UV-visible spectroscopy reader at 520 nm (42–44). All of the experimental results were processed by GraphPad Prism 6.0.

### Real-time quantitative PCR.

For D39-WT and D39Δ*spd1495*, total RNA was extracted with TRIzol reagent (Invitrogen) according to the manufacturer’s protocol following treatment with 100 mg/ml lysozyme. The RNA concentration was determined by using a NanoDrop 2000 UV-VIS spectrophotometer (Thermo Fisher Scientific). A 1-μg mRNA sample was reverse transcribed using a transScript one-step gDNA removal and cDNA synthesis SuperMix kit for qPCR (TransGen Biotech, China) according to the manufacturer’s instructions. RT-qPCR was performed using the StepOne system (Applied Biosystems, USA) with the TransStart Tip Green qPCR supermix kit (TransGen Biotech); 16S RNA was used as an internal control. The cycle threshold (*C_T_*) value was recorded, and the relative quantification of gene expression was calculated using the 2^–ΔΔ^*^CT^* method (45). The results are presented as the gene expressions in the *spd1495* mutant against those in the D39-WT strain. All data were obtained from three independent biological experiments. The primers used for RT-qPCR are shown in Table 4.

### Animal experiments.

All of the animals used in this study were purchased from the Department of Experimental Animal (Beijing HFK Bioscience); animal experiments were approved by the Ethics Committee for Animal Experiments of Jinan University. The 6- to 8-week-old female BALB/c mice were cared for according to the institutional guidelines for animal care under standard conditions.

For mouse infections, D39-WT, D39Δ*spd1495*, and D39*spd1495+* mutants were grown in THY medium and harvested in the exponential growth phase. The bacteria were resuspended in 0.01 M PBS and adjusted to the concentration of 1 × 10^7^ CFU/50 μl. Then, 50-μl portions of the D39-WT, D39Δ*spd1495*, and D39*spd1495+* strains were injected into 6- to 8-week-old female BALB/c mice (*n* = 8) via tail vein injection. The survival of the mice was recorded every 12 h for 4 days.

### Coimmunoprecipitation assay.

D39-WT in the exponential growth phase was harvested by centrifugation at 10,000 × *g* for 10 min at 4°C and washed three times with 0.01 M PBS. D39-WT was resuspended in fresh cell lysates (1 mM phenylmethylsulfonyl fluoride, 50 mM Tris HCl [pH 8.0], 150 mM NaCl, 1% NP-40) until the cell lysates were fully dissolved. The supernatant was collected by centrifugation at 10,000 × *g* for 10 min at 4°C and then incubated with 40 μl of protein A/G-agarose (GE Healthcare, USA) and 5 μl of anti-mouse IgG for 1 h at 4°C, respectively, to remove the nonspecific binding protein, which can bind to IgG, followed by centrifugation at 2,500 × *g* for 5 min at 4°C. Protein A/G-agarose was added, and the supernatants were incubated again. After centrifugation, the concentrations of supernatants were measured by a BCA protein assay kit. SPD_1495 antibody (2 μg) and IgG (2 μg) were added to the supernatants, followed by incubation for 16 h at 4°C, respectively. Then, 40 μl of protein A/G-agarose was added to bind the proteins at 4°C for 6 h, which were collected by centrifugation at 2,500 × *g* for 5 min at 4°C and then resuspended in 800-μl portions of lysates. The isolated proteins were separated by SDS-PAGE, was stained by AgNO_3_, and then digested and identified by mass spectrometry (LTQ Orbitrap XL; Thermo Fisher Scientific).

### Surface plasmon resonance.

For SPR analysis, SPD_1495 protein (30 μg/ml) was fixed on a NanoGold-COOH sensor chip by capture coupling, and then recombinant ComE and ComE^D58E^ in 0.01 M PBS running buffer were injected sequentially into the chamber at different concentrations (50, 100, 200, and 400 nmol/liter for ComE and 100, 200, 400, 800, and 1,600 nmol/liter for ComE^D58E^). The interaction of SPD_1495 with ComE was detected by using Open-SPR (Nicoya, Canada) at 25°C. The binding time and disassociation time were both 300 s, the flow rate was 20 μl/s, and the chip was regenerated with hydrochloric acid (pH 2.5). A one-to-one diffusion-corrected model was fitted to the wavelength shifts corresponding to the various protein concentrations. The data were retrieved and analyzed by using TraceDrawer software.

### Electrophoretic mobility shift assay.

For EMSA, the 218-bp upstream promoter region of the *cps* gene cluster was amplified by PCR (for the primers, see Table 4) to produce the 5ʹ-biotin-labeled probe Bio-*cps* from D39-WT genomic DNA. The amplified fragment was purified by gel extraction. EMSA was carried out according to the LightShift R chemiluminescent EMSA kit protocol (Thermo Fisher Scientific). Proteins of interest (2, 4, 6, or 8 μg) and 100 ng of labeled probe were incubated at 37°C for 30 min in 20 μl of reaction buffer, including 1×binding buffer, 1 μg of poly(dI-dC), 2.5% glycerol, 0.05% NP-40, and 5 mM MgCl_2_; the *cps* without binding protein was used as a negative control. After incubation, electrophoresis in 6% native Tris-borate-EDTA polyacrylamide gels was performed at 100 V for 60 min to analyze the binding reactions. The polyacrylamide gels were transferred to nylon membranes at 380 mA for 40 min. The membranes were cross-linked for 10 min using a UV lamp equipped with 254-nm bulbs, and subsequently biotin-labeled DNA was detected by chemiluminescence according to supplier’s instructions (Thermo Fisher Scientific).

### Evolutionary analysis.

The amino acid sequence of SPD_1495 from S. pneumoniae D39 was used to query the NCBI database using the protein BLAST tool. The multiple sequence alignment and cluster analysis of the high-scoring proteins were performed by the software package Clustal-X 2.1. MEGA5.05 was applied to construct the protein evolutionary tree.

### Statistics.

Data were analyzed by two-tailed, unpaired Student *t* tests, and are expressed as means ± the standard deviations (SD). Statistical analysis was conducted using Prism 6.0 (GraphPad Software, USA). Results were considered significant at *P* < 0.05.

### Data availability.

The raw proteomic data and search results have been deposited to the ProteomeXchange Consortium via the PRIDE (46) partner repository and can be accessed with the reviewer account (website, http://www.ebi.ac.uk/pride; username, reviewer70538@ebi.ac.uk; password, x5Zecn7h).
