# Supplementary material for: SPD_1495 Contributes to Capsular Polysaccharide Synthesis and Virulence in Streptococcus pneumoniae
Source: mSystems. 2020 Feb 25;5(1):e00025-20. doi: 10.1128/mSystems.00025-20 (PMC7043342; doi:10.1128/mSystems.00025-20)
Supplement: TABLE S1 [file mSystems.00025-20-st001.pdf]

Accession	Description	Replication 1			Replication 2			AVERAGE(116:114 fold-changes)	AVERAGE(P-value)	Protein Function
		%Cov	116:114	PVal 116:114	%Cov	116:114	PVal 116:114			
A0A0H2ZL68	Sugar ABC transporter, sugar-binding	50.68	0.034995	1.13E-05	63.57	0.042073	5.15E-10	0.038534	5.65E-06	Uncharacterized
A0A0H2ZM73	6-phospho-beta-glucosidase	45.19	9.908319	5.11E-06	61.72	5.19996	2.05E-05	7.5541395	1.28E-05	scopolin beta-glucosidase activity
A0A0H2ZM91	Cell division protein DivIVA	68.7	0.16293	0.019542	88.55	0.483059	0.044228	0.3229945	3.19E-02	Cell division protein
A0A0H2ZM98	CTP synthase	59.07	0.255859	6.22E-06	66.54	0.299226	2.95E-09	0.2775425	3.11E-06	CTP synthase activity
A0A0H2ZMA2	ATP-dependent zinc metalloprotease FtsH	62.58	0.496592	0.008145	68.25	0.444631	0.0006	0.4706115	4.37E-03	ATPase activity
A0A0H2ZMB9	ATP-dependent Clp protease, ATP-binding subunit	77.46	15.84893	1.35E-13	73.75	11.27197	8.02E-09	13.56045	4.01E-09	ATP binding and peptidase activity
A0A0H2ZMF9	Penicillin-binding protein 2A	51.44	0.539511	0.046644	56.09	0.452898	0.016331	0.4962045	3.15E-02	transferase activity
A0A0H2ZMG6	Uncharacterized protein	36.67	8.317637	0.006966	35.33	9.549926	0.001579	8.9337815	4.27E-03	Uncharacterized
A0A0H2ZMI1	Beta-galactosidase, putative	53.1	5.495409	0	56.69	3.10456	3.15E-01	4.2999845	1.58E-01	hydrolase activity
A0A0H2ZMS8	Sugar isomerase domain protein AgaS	45.88	4.528976	4.78E-06	47.68	1.870682	0.026287	3.199829	1.31E-02	isomerase activity
A0A0H2ZNG6	ATP-dependent Clp protease ATP-binding subunit ClpE	68.35	1.819701	4.35E-05	69.68	1.786488	0.001713	1.8030945	8.78E-04	ATP binding and peptidase activity
A0A0H2ZNM6	Trans-2-enoyl-ACP reductase II	61.42	0.135519	2.91E-05	69.44	0.190546	0.000166	0.1630325	9.76E-05	nitronate monooxygenase activity
A0A0H2ZNN4	Glyceraldehyde-3-phosphate dehydrogenase, NADP-dependent	68.78	2.070141	0.00072	61.18	2.290868	6.28E+00	2.1805045	3.14E+00	glyceraldehyde-3-phosphate dehydrogenase activity
A0A0H2ZNR2	D-alanine transfer protein	51.18	0.608135	0.025307	54.74	0.325087	7.57E-05	0.466611	1.27E-02	lipoteichoic acid biosynthesis
A0A0H2ZNS9	Amino acid ABC transporter, amino acid-binding protein/permease protein	52.59	0.366438	0.006465	55.47	0.47863	0.000248	0.422534	3.36E-03	transmembrane transporter activity
A0A0H2ZNW3	Cmp-binding-factor 1	64.67	0.328095	0.005208	73.82	0.416869	0.016454	0.372482	1.08E-02	Uncharacterized
A0A0H2ZP11	PTS system IIA component, putative	43.67	6.668067	0.036802	43.67	3.250873	0.041979	4.95947	3.94E-02	kinase activity
A0A0H2ZP43	Hydrolase, haloacid dehalogenase-like family protein	51.36	5.19996	0.020728	66.93	7.943282	9.59E-05	6.571621	1.04E-02	hydrolase activity
A0A0H2ZP48	Tagatose 1,6-diphosphate aldolase	76.99	3.837072	6.36E-05	78.83	1.940886	0.000596	2.888979	3.30E-04	hydrolase activity
A0A0H2ZPF7	Glycosyl transferase, group 2 family protein	49.04	9.37562	1.34E-06	54.14	12.35947	2.63E-06	10.867545	1.99E-06	tagatose-6-phosphate kinase activity<br>tagatose-bisphosphate aldolase activity
A0A0H2ZPL7	Uncharacterized protein	49.47	0.220801	0.016366	48.75	0.331131	0.01312	0.275966	1.47E-02	lipid binding activity
A0A0H2ZPN9	Amino acid ABC transporter, amino acid-binding protein/permease protein	48.13	0.570164	0.035213	51.87	0.469894	0.018214	0.520029	2.67E-02	ligand-gated ion channel activity
A0A0H2ZPZ1	Alcohol dehydrogenase, zinc-containing	66.08	2.630268	0.000174	79.06	3.019952	2.62E-05	2.82511	1.00E-04	zinc ion binding and alcohol dehydrogenase activity
A0A0H2ZQ08	Glucan 1,6-alpha-glucosidase	45.05	16.59587	0.000114	48.6	21.87762	1.13E-06	19.236745	5.76E-05	glucan 1,6-alpha-glucosidase activity
A0A0H2ZQA2	Uncharacterized protein	61.72	0.201372	2.88E-05	64.11	0.428549	0.003583	0.3149605	1.81E-03	Uncharacterized
A0A0H2ZQD3	Glycosyl transferase, group 1 family protein	48.56	8.016781	0.006329	58.33	9.120109	1.42E-07	8.568445	3.16E-03	transferase activity, transferring glycosyl groups
A0A0H2ZQD7	Serine protease	62.09	0.343558	0.000568	80.15	0.394457	9.97E-05	0.3690075	3.34E-04	serine-type endopeptidase activity
A0A0H2ZQK8	ABC transporter, substrate-binding protein	65.58	5.597576	4.75E-05	65.17	2.421029	1.07E-06	4.0093025	2.43E-05	ABC transporter, substrate-binding activity
A0A0H2ZQS9	DhaL domain-containing protein	54.05	0.376704	0.004019	57.12	0.487529	0.011465	0.4321165	7.74E-03	glycerone kinase activity
A0A0H2ZR50	ABC transporter, substrate-binding protein	81.52	0.390841	0.002025	80.07	0.387258	0.008047	0.3890495	5.04E-03	ABC transporter, substrate-binding activity
A0A0H2ZRF9	Dihydroorotate dehydrogenase	38.14	9.549926	0.001921	46.15	4.405549	0.000145	6.9777375	1.03E-03	Catalyzes the conversion of dihydroorotate to orotate
A0A0H2ZRL6	PTS system, trehalose-specific IIABC components	21.53	0.288403	0.026287	28.55	0.457088	0.010862	0.3727455	1.86E-02	kinase activity and<br>protein-N(PI)-phosphohistidine-sugar phosphotransferase activity and<br>trehalose transmembrane transporter activity
Q04HT9	ATP synthase subunit beta	77.99	0.380189	0.000646	78.63	0.331131	0.000581	0.35566	6.14E-04	proton-transporting ATP synthase activity
Q04IN8	Pneumolysin	75.8	1.570363	0.016355	87.69	2.128139	0.000925	1.849251	8.64E-03	cholesterol binding and<br>toxin activity
Q04J36	Ribonuclease Y	75.05	0.586138	0.002572	73.56	0.394457	4.51E-05	0.4902975	1.31E-03	endoribonuclease activity
Q04JH1	Adenine phosphoribosyltransferase	74.12	0.487529	0.034372	87.06	0.554626	0.032173	0.5210775	3.33E-02	adenine phosphoribosyltransferase activity
Q04JN5	Pyridoxal 5'-phosphate synthase subunit	61.86	2.398833	0.004621	63.57	2.089296	0.00391	2.2440645	4.27E-03	pyridoxal 5'-phosphate synthase (glutamine hydrolysing) activity
Q04K45	Bifunctional protein PyrR	80.92	4.655861	1.15E-05	83.24	2.728978	9.27E-05	3.6924195	5.21E-05	RNA binding and uracil phosphoribosyltransferase activity
Q04K46	Aspartate carbamoyltransferase	62.21	4.786301	6.77E-06	73.62	2.558586	5.33E-05	3.6724435	3.00E-05	amino acid binding and aspartate carbamoyltransferase activity
Q04K48	Carbamoyl-phosphate synthase large chain	55.86	5.445026	0	66.45	3.372873	0	4.4089495	0.00E+00	TP binding and carbamoyl-phosphate synthase (glutamine-hydrolyzing) acti
Q04KC2	Galactose-6-phosphate isomerase subunit LacB	56.14	5.861382	0.003944	66.08	1.786488	0.02255	3.823935	1.32E-02	galactose-6-phosphate isomerase activity

Q04KU8	Foldase protein PrsA	84.35	0.387258	1.84E-05	87.54	0.322107	1.95E-06	0.3546825	1.02E-05	peptidyl-prolyl cis-trans isomerase activity
Q04LW0	Translation initiation factor IF-2	74.84	0.409261	0.000725	76.34	0.405508	0.000199	0.4073845	4.62E-04	GTPase activity and translation initiation factor activity
Q04MA3	dTDP-glucose 4,6-dehydratase	58.74	18.87991	4.61E-08	59.31	15.13561	1.55E-08	17.00776	3.08E-08	dTDP-glucose 4,6-dehydratase activity
Q04MC1	UPF0371 protein SPD_0310	51.82	9.817479	2.19E-07	51.42	27.54229	1.56E-08	18.6798845	1.17E-07	
Q04MC2	S-ribosylhomocysteine lyase	77.5	14.85936	0.000664	38.12	23.33458	0.002304	19.09697	1.48E-03	iron ion binding and S-ribosylhomocysteine lyase activity
Q9ZIH5	Cps2M	61.42	10.86426	1.93E-06	64.47	13.06171	0.010862	11.962985	5.43E-03	dTDP-4-dehydrorhamnose 3,5-epimerase activity
Q9ZIH6	Glucose-1-phosphate thymidylyltransferase	66.78	10.96478	2.31E-07	73.36	10.76465	1.72E-11	10.864715	1.16E-07	glucose-1-phosphate thymidylyltransferase activity and metal ion bindin
Q9ZIH8	Cps2K	74.76	17.06082	0	84.47	12.24616	0	14.65349	0.00E+00	NAD binding and UDP-glucose 6-dehydrogenase activity
Q9ZII0	Cps2I	56.88	5.495409	0.00011	63.12	8.790225	0.010862	7.142817	5.49E-03	transferase activity, transferring glycosyl groups
Q9ZII4	Cps2T	61.68	10.28016	5.39E-07	69.04	13.55189	8.28E-09	11.916025	2.74E-07	transferase activity, transferring glycosyl groups
Q9ZII5	Cps2E	32.09	5.248075	0.011878	33.63	7.046931	0.000891	6.147503	6.38E-03	transferase activity
Q9ZII6	Cps2D	43.81	4.570882	0.034892	53.54	4.74242	0.029039	4.656651	3.20E-02	kinase activity
Q9ZII7	Chain length determinant protein/polysaccharide export protein, MPA1 family protein	60	8.165824	2.47E-05	60.87	10.18591	3.76E-06	9.175867	1.42E-05	carbohydrate:proton symporter activity
